# Pharmacokinetic Optimization of Radiocopper-Based Theranostic Pretargeting

**DOI:** 10.1021/acs.molpharmaceut.5c01512

**Published:** 2026-01-28

**Authors:** Mike A. Cornejo, Zachary V. Samuels, Gina Dehlavi, Lukas Carter, Wei-Siang Mark Kao, Emilia Strugala, Brian M. Zeglis

**Affiliations:** † Department of Chemistry, Hunter College, 5924 The City University of New York, New York, New York 10021, United States; ‡ Ph.D. Program in Chemistry, Graduate Center of the City University of New York, New York, New York 10016, United States; § Department of Radiology, Memorial Sloan Kettering Cancer Center, New York, New York 10065, United States; ∥ Ph. D.Program in Biochemistry, Graduate Center of the City University of New York, New York, New York 10016, United States; ⊥ Department of Medical Physics, 5803 Memorial Sloan Kettering Cancer Center, New York, New York 10065, United States; # Department of Radiology, Weill Cornell Medical College, New York, New York 10021, United States

**Keywords:** pretargeting, multistep targeting, positron emission tomography, PET, radiopharmaceutical therapy, targeted radionuclide therapy, radioimmunotherapy, pretargeted radioimmunotherapy, radiocopper, copper-64, copper-67, sarcophagine, PEGylation

## Abstract

In vivo pretargeting offers a strategy to improve nuclear imaging and radiopharmaceutical therapy by increasing tumor-to-background activity concentration ratios and decreasing radiation burden to healthy tissues. One particularly promising approach to in vivo pretargeting is predicated on the inverse electron-demand Diels–Alder (IEDDA) ligation between tetrazine (Tz)-based radioligands and *trans*-cyclooctene (TCO)-bearing immunoconjugates. Not surprisingly, the performance of such systems is highly dependent upon the pharmacokinetic profiles of the small molecule radioligands. Herein, we report the synthesis and characterization of a trio of sarcophagine-bearing tetrazines—SarAr-Tz, SarAr-PEG_5_-Tz, and SarAr-PEG_10_-Tz—as well as their radiolabeling with copper-64 (^64^Cu, *t*_1/2_ ∼ 12.7 h), a positron-emitting radioisotope of copper. These radioligands were paired with a TCO-bearing variant of the A33 antigen-targeting antibody huA33 (i.e., huA33-TCO) for pretargeted immunoPET in a murine model of colorectal cancer, revealing that all three produced images with excellent tumor-to-background contrast, but [^64^Cu]­Cu-SarAr-PEG_10_-Tz yielded the best tumor-to-tissue activity concentration ratios. In light of its superior performance, SarAr-PEG_10_-Tz was subsequently radiolabeled with copper-67 (^67^Cu, *t*_1/2_ ∼ 61.8 h), a β^–^-emitting radioisotope of copper, to produce [^67^Cu]­Cu-SarAr-PEG_10_-Tz. This radioligand was then paired with huA33-TCO for in vivo biodistribution and longitudinal therapy studies, ultimately revealing that pretargeted radioimmunotherapy with [^67^Cu]­Cu-SarAr-PEG_10_-Tz exhibits promising efficacy and safety in a murine model of colorectal cancer.

## Graphical abstract

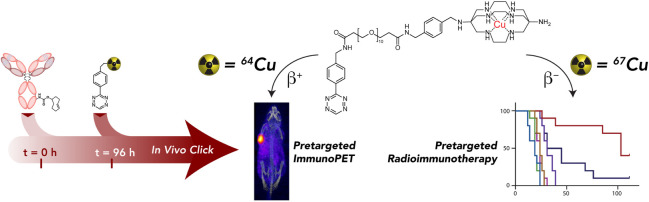

## Introduction

Over the last three decades, radiolabeled monoclonal antibodies (mAb) have emerged as powerful tools in oncology. Indeed, a wide variety of mAb labeled with radionuclides that emit γ-rays, (e.g., ^111^In), positrons (e.g., ^89^Zr, ^124^I), β^–^-particles (e.g., ^177^Lu, ^131^I), or α-particles (e.g., ^225^Ac) have been translated to the clinic for the nuclear imaging and radiopharmaceutical therapy of cancer. Among these, ^89^Zr-girentuximab, ^131^I-omburtumab, ^89^Zr-pertuzumab, ^177^Lu-trastuzumab, ^225^Ac-Actimab-A offer particularly compelling recent examples.^1–7^ Despite this promise, the multiday pharmacokinetic profiles of full-length antibodies can create high radiation doses to healthy tissues, a phenomenon that can be particularly problematic—and, in some cases, clinically disqualifying—in the context of radioimmunotherapy (RIT).^8,9^

In vivo pretargeting is an elegant method designed to circumvent concerns about the pharmacokinetics and dosimetry of radiolabeled antibodies.^10–12^ Pretargeting is predicated on a counterintuitive choice: decoupling the radioactivity from the antibody. The antibody is injected first and allowed time (usually several days) to accumulate in the target tissue and clear from the blood. Subsequently, the radioactivity—typically as part of a small molecule radioligand—is administered; this moiety travels through the body quickly, combining with the tumor-bound immunoconjugate or clearing rapidly from circulation. Over the years, scientists have turned to several technologies to facilitate the in vivo ligation between the antibody and the radioligand, including biotin and streptavidin, bispecific antibodies, complementary oligonucleotides, and host–guest chemistry.^11^ Recently, our team and others have turned to bioorthogonal click chemistry, specifically the extraordinarily rapid inverse electron-demand Diels–Alder reaction between tetrazine (Tz) and *trans*-cyclooctene (TCO), to facilitate pretargeting.^13–16^ To this end, a TCO-bearing immunoconjugate is administered intravenously, followed days later by a Tz-based radioligand that either clicks with the mAb-TCO at the tumor or clears from the body (Figure 1). This approach has been validated in preclinical models for both imaging and therapy using an array of antibodies and radionuclides ranging from ^11^C to ^225^Ac.^17–19^

**Figure 1:**
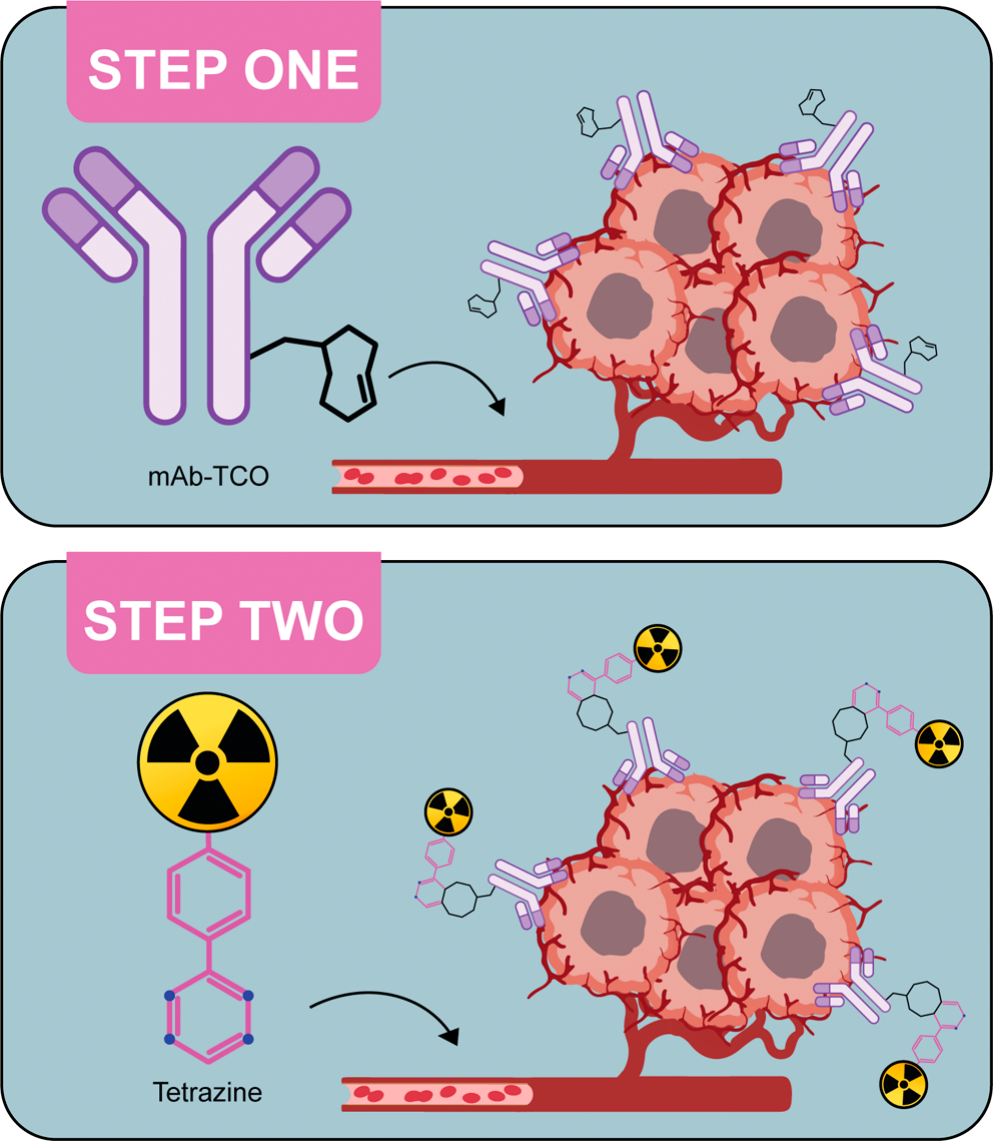
Schematic of in vivo pretargeting. The mAb-TCO is administered first and allowed to accumulate at the target site and clear from the blood. Then, a radiolabeled tetrazine is administered that travels quickly through the body, either clicking to the tumor-bound immunoconjugate to enable imaging or therapy or clearing from the blood. This figure was created with BioRender.

We have previously leveraged TCO-bearing antibodies and a sarcophagine-bearing Tz radioligand (SarAr-Tz) to facilitate ^64^Cu-based pretargeted PET imaging in murine models of colorectal and pancreatic cancer.^20^ These systems yielded excellent tumor-to-background contrast, and one pairing—[^64^Cu]­Cu-SarAr-Tz and 5B1-TCO—is the subject of an active clinical trial focused on the pretargeted PET of CA19-9-expressing cancers at Memorial Sloan Kettering Cancer Center (NCT05737615). In light of this promise, we labeled the Tz-bearing ligand with ^67^Cu, a β^–^-emitting radioisotope of copper with a half-life of 2.6 d, and subsequently combined [^67^Cu]­Cu-SarAr-Tz with a TCO-bearing variant of the A33-targetng mAb huA33 (i.e., huA33-TCO) for pretargeted radioimmunotherapy (PRIT) in a murine model of colorectal cancer.^21^ While this system provided promising safety and efficacy, the in vivo performance of pretargeting systems is highly dependent on the pharmacokinetic profile of the radioligand. Indeed, our previous research has shown that relatively minor changes to the structure of radioligands can dramatically alter their uptake in the tumor, tumor-to-background contrast, and therapeutic indices. Herein, we describe our work toward the creation of an optimized sarcophagine-tetrazine radioligand for radiocopper-based pretargeted imaging and therapy.

## Methods and Materials

### General

All reagents were purchased from Fisher Scientific unless otherwise stated. HuA33 was obtained from Genscript, Inc. (Piscataway, New Jersey), and the SW1222 cell line was purchased from ATCC (Manassas, Virginia). TCO-NHS, Tz-NHS, and Tz-PEG_5_-NHS ester were purchased from VectorLabs (Newark, California), and HOOC-PEG_10_-NH_2_ was obtained from BroadPharm (San Diego, California). Copper-64 was purchased from Washington University, St. Louis as [^64^Cu]­CuCl_2_ in 0.05 M HCl. Copper-67 was purchased from the Idaho Accelerator Center (Idaho State University, Pocatello, Idaho, USA) as [^67^Cu]­CuCl_2_ in 0.01 M HCl. All experiments involving laboratory animals were performed using protocols approved by the institutional animal care and use committees (IACUC) of Weill Cornell Medical College and Hunter College (Protocol #2015-0004).

All instruments were calibrated and maintained according to standard quality control practices and procedures. All high-performance liquid chromatography (HPLC) was performed using a Shimadzu HPLC system and a C_18_ reverse phase Phenomenex Jupiter column (5 μm, 10 × 250 mm) or a Superdex 200 Increase 10/300 GL column. UV–vis measurements were taken on a Thermo Scientific NanoDrop One Microvolume UV–vis Spectrophotometer (Thermo Fisher Scientific, Fair Lawn, NJ). Radioactivity was measured using a CRC-15R Dose Calibrator (Capintec, Inc., Ramsey, NJ), and biodistribution samples were counted on a calibrated Automatic Wizard^2^ γ-counter (PerkinElmer, Inc., Waltham, MA). The labeling of the radioligand was monitored using glass-fiber, silica-impregnated instant thin-layer chromatography (iTLC) paper (Pall Corp., East Hills, NY) and analyzed on an AR-2000 radio-TLC plate reader using Winscan Radio-TLC software (Bioscan, Inc., Washington, DC.

### Synthesis of SarArTz (1)

In a small glass vial, 10 mg of SarAr-NH_2_ were dissolved in 400 μL of extra dry dimethylformamide. Subsequently, 1.5 eq. of Tz-NHS was added along with 1.5 eq. of *N,N*-diisopropylethylamine (DIPEA). The mixture was stirred for 1 h at room temperature, and the product was purified via semipreparative C_18_–HPLC using a gradient of 5–95% MeCN/Water +0.1% TFA over 30 min (t_R_ = 11.8 min). Exact mass: 631.42; observed mass: [M + H]^+^= 632.42, [M+2H]^2+^= 316.87.

### Synthesis of SarAr-PEG_5_-Tz (2)

In a small glass vial, 10 mg of SarAr-NH_2_ were dissolved in 400 μL of extra dry dimethylformamide. Subsequently, 1.5 eq. of Tz-PEG_5_-NHS was added along with 1.5 eq. of *N,N*-diisopropylethylamine (DIPEA). The mixture was stirred for 1 h at room temperature, and the product was purified via semipreparative C_18_–HPLC using a gradient of 5–95% MeCN/Water +0.1% TFA over 30 min (*t*_R_ = 12.0 min). Exact mass: 922.59; observed mass: [M + H]^+^= 923.64, [M+2H]^2+^= 461.82.

### Synthesis of SarAr-PEG_10_-Tz (3)

In a small glass vial, 10 mg of Tz-PEG_10_-COOH were dissolved in 600 μL of dimethylformamide, and 3 eq. of DIPEA were added. After 10 min, 1.2 eq. of HATU were added, and the mixture was stirred at room temperature for 1 h. Then, 1.3 eq. of SarAr-NH_2_ were mixed in the vial, and the reaction was stirred overnight at room temperature. The product was purified via semipreparative C_18_–HPLC using a gradient of 5–95% MeCN/Water +0.1% TFA over 30 min (*t*_R_ = 12.5 min). Exact mass: 1142.72; observed mass: [M + H]^+^= 1143.64, [M+2H]^2+^= 571.82.

### Cell Culture

The human colorectal cell line SW1222 was obtained from ATCC and maintained in Iscove’s Modified Dulbecco’s Medium (IMDM) supplemented with 10% heat-inactivated fetal calf serum, 2 mM glutamine, 100 units/mL penicillin, and 100 units/mL streptomycin in a 37 °C environment containing 5% CO_2_. The cell line was harvested and passaged every 5 days using 0.25% trypsin/0.53 mM EDTA in Hank’s Buffered Salt Solution without calcium and magnesium. All media was purchased from the Media Preparation Facility at Memorial Sloan Kettering Cancer Center.

### Radiolabeling with ^64^Cu and ^67^Cu

A solution of 7 μg of SarAr-Tz, SarAr-PEG_5_-Tz, or SarAr-PEG_10_-Tz was prepared in NH_4_OAc buffer (0.25 M, pH 5.5, 200 μL). Then, [^64^Cu]­CuCl_2_ or [^67^Cu]­CuCl_2_ in 0.05 M HCl (185–370 MBq) was added to the reaction mixture, and it was incubated on a thermomixer at 600 rpm for 15 min at 37 °C. After incubation, radio-instant thin layer chromatography (iTLC) using an eluent of 50 mM EDTA (pH 5.5) was performed to determine the radiochemical yield of the reaction.

### Partition Coefficients

A 1:1 mixture of phosphate buffer solution (PBS, pH 7.4) and 1-octanol was prepared in 8 mL glass vials. 0.037 MBq of [^64^Cu]­Cu-SarAr-Tz, [^64^Cu]­Cu-SarAr-PEG_5_-Tz, or [^64^Cu]­Cu-SarAr-PEG_10_-Tz was added to the mixture and vortexed vigorously for 5 min. Then, 1 mL of each layer was transferred to small vials, and the amount of radioactivity was counted on a gamma counter calibrated for ^64^Cu. The following formula was used to calculate the partition coefficients:

$$\log D_{7.4}=\log_{10}\left[\frac{\mathrm{count}s_{\mathrm{octanol}}}{\mathrm{count}s_{\mathrm{PBD}}}\right]$$

### Bioconjugation of huA33-TCO

A solution of 1 mg huA33 in 500 μL PBS (pH 7.4) was placed in a LoBind Eppendorf microcentrifuge tube, and the pH was then increased to 9.5 via the addition of small aliquots of 0.1 M of sodium bicarbonate. Subsequently, 80 eq. of TCO-NHS in 500 μL DMSO were added, and the resulting solution was allowed to react on a thermomixer for 1 h at RT and 500 rpm. The mixture was purified using size exclusion chromatography (Sephadex G-25 M, PD-10 column, GE Healthcare; dead volume: 2.5 mL, eluted with 2 mL of Chelex PBS, pH 7.4) and concentrated using centrifugal filtration units with a 50,000 Da molecular weight cutoff (Amicon Ultra 2 mL Centrifugal Filtration Units, MilliporeSigma Corp., Burlington, MA). Size exclusion high-performance liquid chromatography (SE-HPLC) was used to characterize the immunoconjugate after modification. To this end, PBS pH 7.4 was used as a mobile phase with a flow rate of 0.75 mL/min on a Superdex 200 Increase 10/300 GL column (Cytiva, Global Life Sciences Solutions USA LLC, Marlborough, MA, USA).

### Degree of Labeling

A solution of 100 μg huA33-TCO in 500 μL of PBS (pH 7.4) was prepared in a 1.5 mL LoBind Eppendorf vial. A 100-fold excess of Cy3-tetrazine was added to the solution and allowed to react for 30 min at RT in a thermomixer at 500 rpm. The fluorophore-modified product was purified via size exclusion using a PD-10 column and an eluent of PBS and then concentrated via centrifugation using a filter with a 50 kDa cutoff. Absorbance measurements were taken at 280 and 532 nm to calculate the degree of labeling (DOL) using the following formula (*A* = absorbance; MW = molecular weight; ε = molar absorptivity):

$$A_{\mathrm{mAB}}=A_{280}-A_{\max}\left(\mathrm{CF}\right)$$

$$\mathrm{DOL}=\frac{\left[A_{\mathrm{maBax}}\times MW_{\mathrm{mAb}}\right]}{\left[\left[\mathrm{mAB}\right]\times \varepsilon_{\mathrm{Cy}3}\right]}$$

### Subcutaneous Xenografts

Six-to-eight-week-old female athymic nude mice were obtained from Jackson Laboratory and allowed to acclimatize for approximately 1 week prior to inoculation. Animals were housed in ventilated cages and given water and food ad libitum. Mice were anaesthetized by inhalation of a 2% isoflurane (Baxter Healthcare, Deerfield, IL)/oxygen gas mixture and xenografted subcutaneously on the right flank with 5 × 10^6^ SW1222 cells in a 150 μL cell suspension of a 1:1 mixture of fresh media: Matrigel (Corning Life Sciences, Corning, NY). The SW1222 tumors reached the ideal size for imaging, biodistribution, and therapy studies (∼100 mm^3^) after approximately two to 3 weeks.

### PET Imaging

PET imaging was conducted on an Inveon PET/CT scanner (Siemens Medical Solutions, Malvern, PA). Tumor-bearing mice were first injected with 100 μg (0.7 nmol) huA33-TCO in 100 μL 0.9% sterile saline via the lateral tail vein. After 96 h, the same mice were then administered [^64^Cu]­Cu-SarAr-Tz, [^64^Cu]­Cu-SarAr-PEG_5_-Tz, or [^64^Cu]­Cu-SarAr-PEG_10_-Tz (11.1 to 12.95 MBq, 0.07 nmol) in 100 μL 0.9% sterile saline via the lateral tail vein. Approximately 5 min prior to the acquisition of PET data, mice were anesthetized by inhalation of a 2% isoflurane/oxygen gas mixture and kept under anesthesia for the duration of the scan. Static scans were recorded at 6, 12, and 24 h after the intravenous administration of ^64^Cu-labeled radioligands. An energy window of 350–650 keV and a coincidence timing window of 3.432 ns were used. Data were sorted into 2-dimensional histograms by Fourier rebinning, and transverse images were reconstructed by ordered subsets expectation maximization (OSEM). The imaging data were normalized to correct for nonuniformity of response of the detector, physical decay of the radionuclide to the time of injection, dead-time count losses, and positron-branching ratio, but no attenuation, scatter, or partial-volume averaging correction was applied. The counting rates in the reconstructed images were converted to activity concentrations (percentage injected dose per gram of tissue [%ID/g]) using a system calibration factor derived from the imaging of a mouse-sized water-equivalent phantom containing ^64^Cu. Images were analyzed with VivoQuant (Invicro).

### Dosimetry

Briefly, the approach to dosimetry described by Carter et al. was used.^22^ To this end, organ-level biodistribution data for [^67^Cu]­Cu-SarAr-PEG_10_-Tz were acquired at 4, 12, 24, 48, 72, and 96 h times postadministration (vide infra) and were used to project radiation absorbed doses to a representative 25 g mouse. For the purposes of estimating activity concentration in the hematopoietically active (red) bone marrow, the activity concentrations in blood were scaled by a factor of 0.36.^23^ Activity clearance in most tissues was generally well-described by a one- or two-phase exponential decay model, which was fit to the data using nonlinear regression. The tumor tissue exhibited marked uptake over the measured time course, and therefore a two-phase exponential with an uptake period followed by a clearance period was used for the tumor. Time-integrated activity was then calculated using the regression-optimized parameters of the fit functions with the analytical expressions for their integrals (to complete decay). Absorbed doses to tumor and all normal organs were estimated using Monte Carlo radiation transport simulations, which utilize the MOBY mouse phantom and PHITS/PARaDIM software.^24,25^ PARaDIM default settings for physical models of radiation transport were used, and sufficient particle histories were simulated to achieve less than 2% statistical relative error in absorbed dose estimates for each organ of the phantom.

### Longitudinal Radiotherapy Study

In the longitudinal therapy studies, three control cohorts, three PRIT cohorts, and one traditional RIT cohort were employed (*n* = 10). The first control cohort received only saline, the second received only huA33-TCO (100 μg, 0.7 nmol, 6 TCO/mAb, in 100 μL sterile saline), and the third received only [^67^Cu]­Cu-SarAr-PEG_10_-Tz (55.5 MBq, 0.7 nmol, in 100 μL sterile saline). All animals in the pretargeting cohorts were administered huA33-TCO (100 μg, 0.7 nmol, 6 TCO/mAb, in 100 μL sterile saline) followed 96 h later by the injection of [^67^Cu]­Cu-SarAr-PEG_10_-Tz (18.5 MBq, 37 MBq, 55.5 MBq, in 100 μL sterile saline). In the RIT cohort, the mice were administered [^67^Cu]­Cu-SarAr-PEG_10_-Tz-TCO-huA33 (18.5 MBq, 100 μg, 0.7 nmol, in 100 μL sterile saline, see Supporting Information for synthetic details). The volumes of the xenografts were monitored twice a week using calipers. All mice were assessed biweekly throughout the study for outward signs of toxicity, including lethargy, loss of appetite, and decreasing body weight. Four possible end points of the study were defined: (i) if the longest dimension of the tumor reached 2 cm^3^; (ii) if the tumor started to hamper the movement of the mouse; (iii) if the tumor became necrotic; and (iv) if the mouse lost more than 10% of its body weight.

### Blood Analysis

Mice within the longitudinal therapy study were anaesthetized with a 2% isoflurane/oxygen gas mixture before collecting 30 μL of blood from the retro-orbital sinus using a microhematocrit capillary tube. The blood was collected in a Minivette POCT collection tube coated with K3 EDTA (Braintree Scientific, Inc., Braintree, MA, USA) and subsequently analyzed with a HemaVet 950 (Drew Scientific, Inc., Miami Lakes, FL, USA).

### Statistical Analysis

Statistical differences were analyzed with GraphPad Prism software (10.6.0v GraphPad Software Inc.; San Diego, CA, USA) via an unpaired, two-tailed Student’s *t* test with a Welch’s correction. *P ≤ 0.05, **P ≤ 0.01, ***P ≤ 0.001, and ****P ≤ 0.0001.

## Results and Discussion

The first step in the investigation was the synthesis and characterization of the molecular components of the system. Our approach to in vivo pretargeting pairs a TCO-modified antibody with a Tz-bearing radioligand, primarily due to the greater in vivo stability of TCO compared to 1,2,4,5-tetrazine (i.e., the variant of Tz that we use). It is important to note, however, that this does not necessarily need to be the case: in recent years, the advent of more reactive TCO and more stable Tz has given rise to several reports in which the “polarity” of the pretargeting system is switched.^26^ For the study at hand, we synthesized and characterized a trio of ligands in which the sarcophagine and Tz are separated by oligoethylene glycol chains of different lengths: SarAr-Tz, SarAr-PEG_5_-Tz, and SarAr-PEG_10_-Tz (Figure 2). The choice of these linkers was driven by several previous observations by our laboratory, specifically (i) PEGylation is an effective approach to modulating the pharmacokinetics of Tz-bearing radioligands; (ii) PEGylated Tz-bearing radioligands generally outperform Tz-bearing radioligands bearing other types of linkers; and (iii) the introduction of PEG_5_- and PEG_10_-containing linkers can materially change the pharmacokinetic profiles of Tz-bearing radioligands.^20,27^ For each construct, an amine-bearing variant of sarcophagine—SarAr-NH_2_—was synthesized as previously described.^20^ Subsequently, SarAr-NH_2_ was combined with Tz-NHS in 500 μL of DMF; the pH of the solution was raised with DIPEA; and the solution was allowed to stir at room temperature for 1 h, ultimately affording SarAr-Tz in 35% yield. A nearly identical procedure was followed with Tz-PEG_5_-NHS to afford SarAr-PEG_5_-Tz in 48% yield. The lack of a commercially available Tz-PEG_10_ synthon necessitated a different route to SarAr-PEG_10_-Tz. Here, H_2_N-PEG_10_-COOH was first incubated with Tz-NHS and DIPEA to yield Tz-PEG_10_-COOH. This compound was then activated with 1.2 eq. of HATU and combined with SarAr-NH_2_ to produce SarAr-PEG_10_-Tz in 30% yield. Each of the three compounds was purified via semipreparative C_18_ HPLC and characterized via ESI-HPLC and ^1^H NMR (Figures S1–S6).

**Figure 2:**
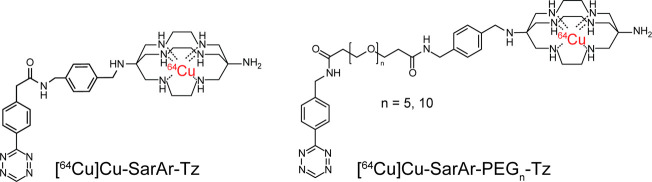
Structures of [^64^Cu]­Cu-SarAr-Tz, [^64^Cu]­Cu-SarAr-PEG_5_-Tz, and [^64^Cu]­Cu-SarAr-PEG_10_-Tz.

The immunoconjugate for this system is based on the huA33 antibody, a humanized IgG_1_ that targets the A33 antigen, a transmembrane gap junction protein expressed in the overwhelming majority of colorectal carcinomas. HuA33 was stochastically modified with TCO-NHS under basic conditions and subsequently purified via gel filtration chromatography, ultimately affording huA33-TCO in ∼80% yield. Size exclusion chromatography confirmed that the immunoconjugate was >98% pure with respect to fragmentation, aggregation, and demetalation. Subsequently, the degree-of-labeling of the immunoconjugate—5.4 ± 0.8 TCO/mAb—was determined via click ligation with a Cy3-bearing variant of Tz followed by UV–vis spectrophotometry (Table S1).

In preparation for in vivo experiments, radiolabeling protocols were developed for each of the ligands. To this end, SarAr-Tz, SarAr-PEG_5_-Tz, and SarAr-PEG_10_-Tz (0.7 nmol) were incubated with [^64^Cu]­CuCl_2_ (74–370 MBq) in 300 μL ammonium acetate buffer (200 mM, pH 5.5) at room temperature. Radio-iTLC and radio-HPLC were employed to monitor the reaction, which reached completion after 20 min. Ultimately, [^64^Cu]­Cu-SarAr-Tz, [^64^Cu]­Cu-SarAr-PEG_5_-Tz, [^64^Cu]­Cu-SarAr-PEG_10_-Tz were isolated *without* subsequent purification in >99% radiochemical conversion, >99% purity, and specific activities of 15–20 MBq/μg (Figure S7). The hydrophilicity (i.e., LogD_7.4_) of each of the radioligands was determined via partition experiments. Values of −2.6 ± 0.1, −3.2 ± 0.1, and −3.3 ± 0.1 were determined for [^64^Cu]­Cu-SarAr-Tz, [^64^Cu]­Cu-SarAr-PEG_5_-Tz, [^64^Cu]­Cu-SarAr-PEG_10_-Tz, respectively, illustrating that the addition of the PEG chains exerts some—but not a tremendous amount of—influence on hydrophobicity. In order to verify the reaction between the click partners, a test ligation was performed between each of the radioligands and huA33-TCO: in each case, size exclusion-HPLC confirmed that the click reaction was complete and quantitative after only 5 min at RT (see Figure S8).

The in vivo evaluation of [^64^Cu]­Cu-SarAr-Tz, [^64^Cu]­Cu-SarAr-PEG_5_-Tz, [^64^Cu]­Cu-SarAr-PEG_10_-Tz began with the determination of the serum half-lives. Along these lines, 11.1–12.9 MBq of each of the radioligands was administered to healthy athymic nude mice via the lateral tail vein, and aliquots of blood were subsequently collected from the contralateral tail vein after 30 s, 1, 2, 5, 10, 30, and 60 min. The activity concentration of the blood at each time point was then determined via gamma counter; the values were plotted as a function of time; and these data were fit to exponential decay curves. This analysis revealed a serum half-life of 31.8 ± 7.9 min for [^64^Cu]­Cu-SarAr-Tz, 28.9 ± 6.4 min for [^64^Cu]­Cu-SarAr-PEG_5_-Tz, and 23.5 ± 5.5 min for [^64^Cu]­Cu-SarAr-PEG_10_-Tz (Figure 3). Interestingly, while the PEGylation of the radioligand seems to have accelerated its clearance from the blood, the effect did not reach the threshold of statistical significance (*p* > 0.05).

**Figure 3:**
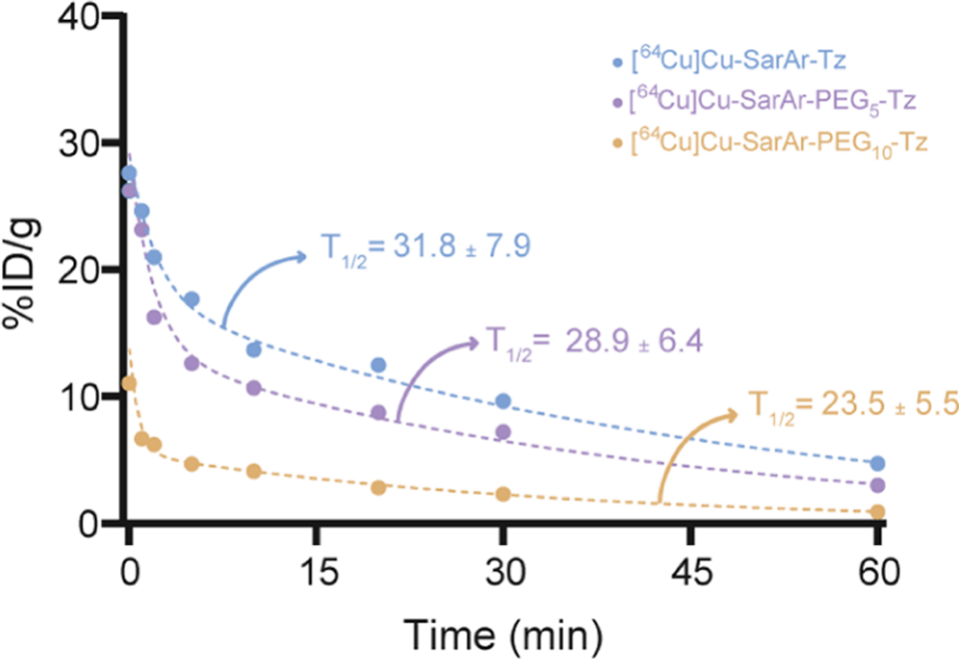
Plot of blood activity concentration vs. time for [^64^Cu]­Cu-SarAr-Tz, [^64^Cu]­Cu-SarAr-PEG_5_-Tz, and [^64^Cu]­Cu-SarAr-PEG_10_-Tz as well as their serum half-life values (in min) derived from the exponential decay curves.

The next step in the investigation was the interrogation of the in vivo performance of the trio of radioligands as tools for in vivo pretargeting. To this end, athymic nude mice bearing SW1222 human colorectal cancer xenografts (*n* = 4 per radioligand) were intravenously administered huA33-TCO (100 μg; 0.7 nmol) followed 96 h later by 11.1 – 12.95 MBq (0.7 nmol) [^64^Cu]­Cu-SarAr-Tz, [^64^Cu]­Cu-SarAr-PEG_5_-Tz, or [^64^Cu]­Cu-SarAr-PEG_10_-Tz. PET images were acquired 6, 12, and 24 h after the administration of the radioligand, and ex vivo biodistribution data were collected immediately after the terminal imaging time point. The PET images clearly illustrate that in vivo pretargeting with all three radioligands effectively delineates tumor tissue with high tumor-to-background contrast, even at the earliest time points (Figure 4; Figure S9). The biodistribution data confirm this observation: the activity concentrations in the tumors at 24 h postinjection were 12.9 ± 2.6%ID/g ([^64^Cu]­Cu-SarAr-Tz), 10.2 ± 0.7%ID/g ([^64^Cu]­Cu-SarAr-PEG_5_-Tz), and 8.7 ± 1.4 ([^64^Cu]­Cu-SarAr-PEG_10_-Tz), and the tumor-to-muscle activity concentration ratios at the same time-point were 27.8 ± 6.6, 23.9 ± 3.1, and 32.6 ± 6.2, respectively (Tables S2–S9). While all three radioligands proved effective, the PET images suggest that pretargeting with [^64^Cu]­Cu-SarAr-PEG_10_-Tz produces lower background uptake. The biodistribution data generally support this notion as well. The starkest differences are—not surprisingly—between the behavior of [^64^Cu]­Cu-SarAr-Tz and [^64^Cu]­Cu-SarAr-PEG_10_-Tz, with [^64^Cu]­Cu-SarAr-PEG_5_-Tz generally lying somewhere in between. Critically, while [^64^Cu]­Cu-SarAr-Tz and [^64^Cu]­Cu-SarAr-PEG_10_-Tz produce comparable activity concentrations in tumor tissue, the latter yields significantly lower activity concentrations in the blood (1.9 ± 0.3%ID/g), lungs (1.6 ± 0.1%ID/g), liver (1.3 ± 0.1%ID/g), spleen (1.0 ± 0.1%ID/g), pancreas (0.4 ± 0.1%ID/g), stomach (0.2 ± 0.0%ID/g), small intestine (0.3 ± 0.0%ID/g), large intestine (0.3 ± 0.1%ID/g), kidneys (2.1 ± 0.2%ID/g), muscle (0.3 ± 0.1%ID/g), and bone (0.5 ± 0.1%ID/g) than the former. While the tumor-to-healthy organ activity concentration ratios for [^64^Cu]­Cu-SarAr-PEG_10_-Tz were not significantly superior to those of [^64^Cu]­Cu-SarAr-Tz, the PEGylated radioligand did produce an improved tumor-to-tissue activity concentration ratio for the stomach.

**Figure 4:**
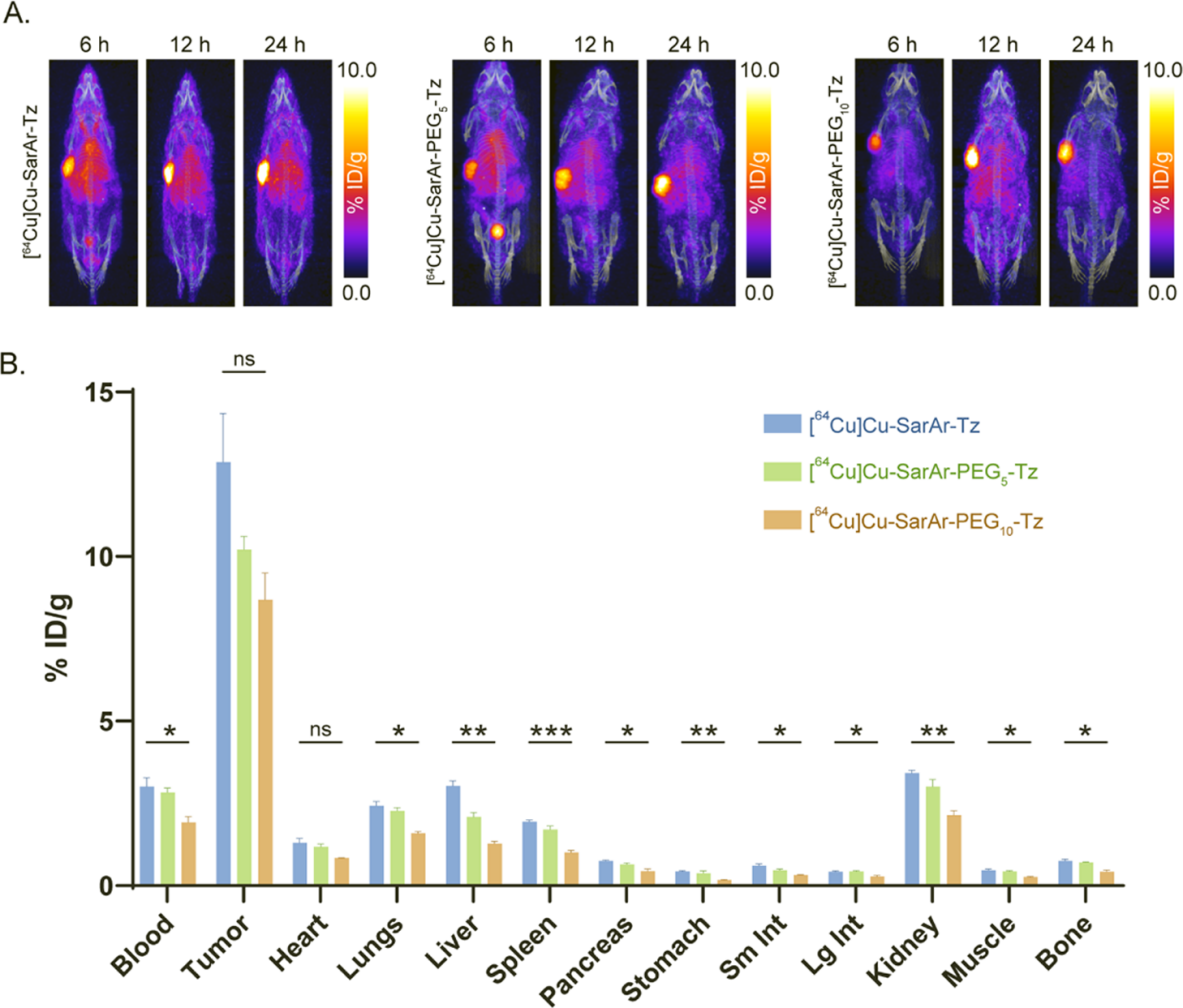
Pretargeted PET and biodistribution data collected from athymic nude mice bearing SW1222 tumors that were administered huA33-TCO followed 96 h later by [^64^Cu]­Cu-SarAr-Tz, [^64^Cu]­Cu-SarAr-PEG_5_-Tz, or [^64^Cu]­Cu-SarAr-PEG_10_-Tz. (A) Maximum intensity projection PET images of a representative mice collected 6, 12, and 24 h after the intravenous administration of the radioligands. (B) Biodistribution data collected immediately after the terminal imaging time point. Statistical analyses shown here were performed between [^64^Cu]­Cu-SarAr-Tz and [^64^Cu]­Cu-SarAr-PEG_10_-Tz. *P* ≤ 0.05*, *P* ≤ 0.01**, *P* ≤ 0.001***, and *P* ≤ 0.0001****, ns = not significant.

While the differences in the in vivo performance of the radioligands were not particularly dramatic, the lower accretion of [^64^Cu]­Cu-SarAr-PEG_10_-Tz in several organs as well as its broadly superior tumor-to-background activity concentration ratios led us to choose this radioligand for subsequent PRIT experiments. To this end, we first optimized the radiolabeling of SarAr-PEG10-Tz with [^67^Cu]­CuCl_2_, ultimately yielding [^67^Cu]­Cu-SarAr-PEG_10_-Tz in >99% radiochemical conversion, >99% purity, and a specific activity of ∼15 MBq/μg. Subsequently, athymic nude mice bearing SW1222 human colorectal cancer xenografts (*n* = 4 per group) were intravenously injected with huA33-TCO (100 μg; 0.7 nmol) followed 96 h later by 11.1–12.9 MBq (0.7 nmol) [^67^Cu]­Cu-SarAr-PEG_10_-Tz, and biodistribution data were collected 4, 12, 24, 48, 72, and 96 h after the administration of the radioligand. The average activity concentration in each organ was then plotted as a function of time and fit to a biexponential function to obtain time-activity curves that in turn were used to calculate the absorbed dose for each organ as well as its therapeutic index (Figure 5). The active bone marrow—with a maximum tolerated dose (MTD) of 2–3 Gy^28^—is the dose limiting organ for most radiopharmaceutical therapy studies; our dosimetry data suggested that the maximal tolerated activity (MTA) for our study would be ∼63 MBq given the absorbed dose coefficient for the marrow of 3.2 cGy/MBq.

**Figure 5:**
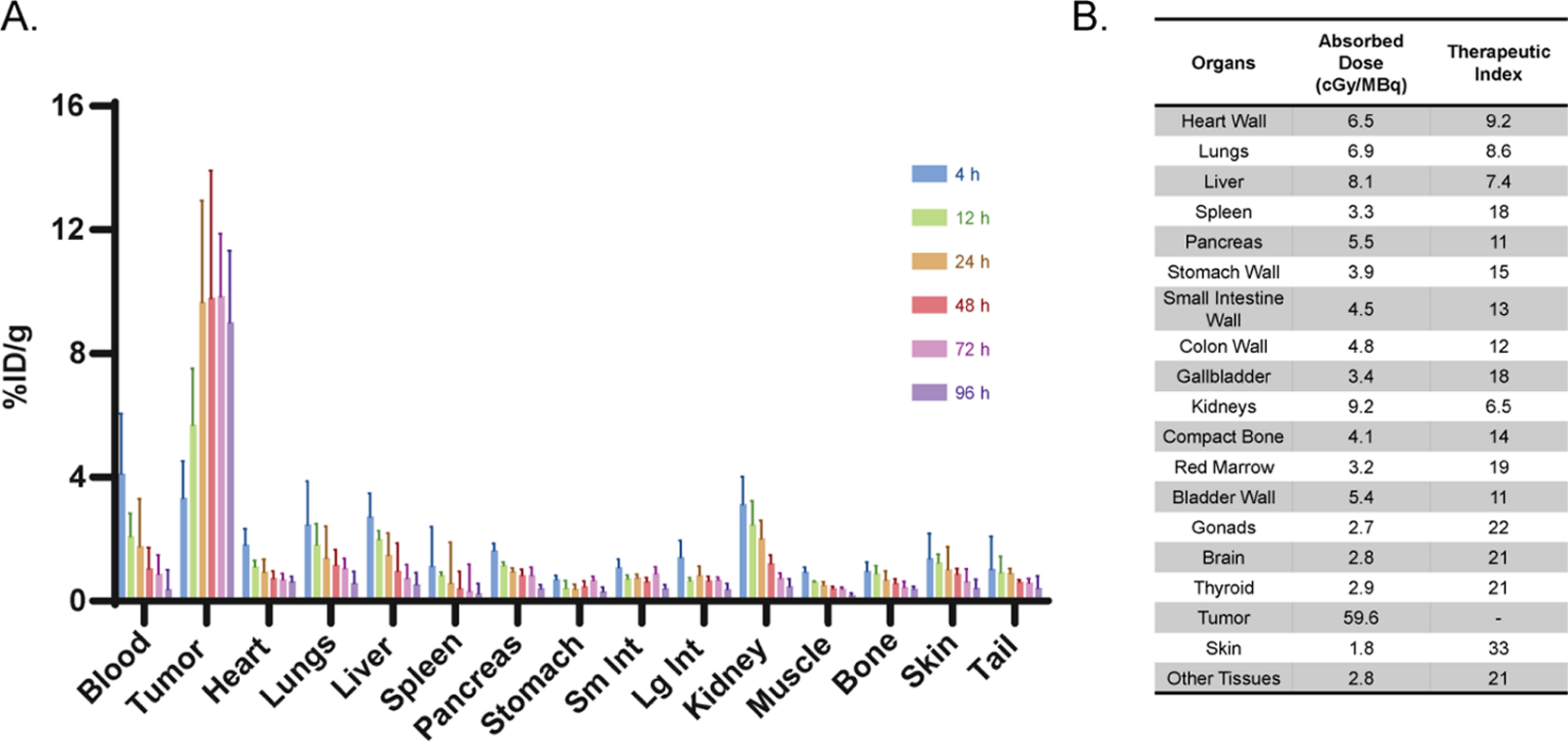
Biodistribution (A) and dosimetry (B) data collected from athymic nude mice bearing SW1222 tumors that were administered huA33-TCO followed 96 h later by [^67^Cu]­Cu-SarAr-PEG_10_-Tz.

The longitudinal therapy study employed seven cohorts of athymic nude mice bearing subcutaneous SW1222 colorectal cancer xenografts (*n* = 10): one that received no treatment at all; one that received only huA33-TCO (100 μg; 0.7 nmol); one that received [^67^Cu]­Cu-SarAr-PEG_10_-Tz alone (55 MBq; 0.7 nmol); three that received huA33-TCO (100 μg; 0.7 nmol) and then, 96 h later, different doses of [^67^Cu]­Cu-SarAr-PEG_10_-Tz (18.5, 37.0, 55.5 MBq; all 0.7 nmol); and one that received [^67^Cu]­Cu-SarAr-PEG_10_-huA33 (18.5 MBq; 100 μg; 0.7 nmol). Critically, only one dose of RIT—18.5 MBq—was used because the literature suggests that higher doses (i.e., 37.0 or 55.5 MBq) could cause radiation toxicity due to the higher radiation dose rates of the directly labeled antibody. Throughout the study, tumor volumes and body weights were measured every 3 d, and blood was collected every 7 d via the retro-orbital sinus for hematoxicological analyses.

The study clearly revealed that pretargeting with huA33-TCO and [^67^Cu]­Cu-SarAr-PEG_10_-Tz exhibits a dose-dependent therapeutic effect (Figure 6). While the tumors of the mice in the control cohorts experienced unchecked growth, those in the trio of huA33-TCO/[^67^Cu]­Cu-SarAr-PEG_10_-Tz cohorts demonstrated inhibited growth, with the effect most evident and dramatic in the cohort that received the highest dose of the radioligand (i.e., 55 MBq). This trend is borne out in the survival data as well. The median overall survival values for the control cohorts were 19 d (saline only), 19 d (huA33-TCO only), and 24 d (55 MBq radioligand only), while those for the pretargeting cohorts were 24 d (18.5 MBq radioligand), 31 d (37 MBq), and 38 d (55 MBq). Interestingly, none of the pretargeting cohorts exhibited as significant a response as did the cohort that received 18.5 MBq of directly radiolabeled [^67^Cu]­Cu-SarAr-PEG_10_-huA33. This is somewhat surprising in light of our previous work on ^67^Cu-PRIT in which the median overall survival of the highest dose PRIT cohort (55 MBq) matched that of RIT cohort (18.5 MBq). However, the aforementioned study used (i) a shorter interval time (i.e., 72 h vs. 96 h here) and (ii) a radioligand (i.e., [^67^Cu]­Cu-MeCoSar-Tz) with no PEG chain and a structure that was similar yet fundamentally different in several ways. The latter makes comparisons between this investigation and these previously collected data difficult; however, the former difference is likely responsible for this phenomenon, as the extra 24 h of pretargeting interval may provide more time for the TCO to isomerize to unreactive *cis-*cyclooctene or for the immunoconjugate to become unavailable for in vivo click ligations (i.e., via deeper penetration of the xenograft or egress from the tumor tissue). Finally, all of the therapeutic regimens tested broadly seemed safe: no significant decreases in the weight of the mice were observed, and all hematoxicological parameters remained at or near healthy physiological levels throughout the experiment. Early drops in WBC and platelets were observed—especially in the higher dose PRIT and RIT cohorts—but these values recovered to pretherapy levels by the end of the experiment.

**Figure 6:**
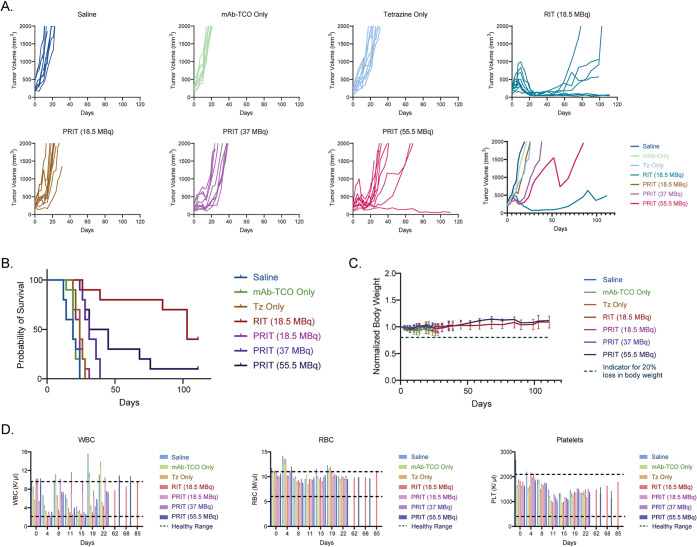
Data from the longitudinal pretargeted radioimmunotherapy study: (A) tumor volumes for each cohort as a function of time; (B) Kaplan–Meier survival curves; (C) normalized body weight as a function of time; (D) white blood cell (WBC), red blood cell (RBC), and platelet counts as a function of time.

## Conclusions

In this investigation, we successfully synthesized, characterized, and validated a trio of radiocopper-labeled ligands for pretargeted radiotheranostics: [^64/67^Cu]­Cu-SarAr-Tz, [^64/67^Cu]­Cu-SarAr-PEG_5_-Tz, and [^64/67^Cu]­Cu-SarAr-PEG_10_-Tz. The addition of the oligoethylene glycol chains altered both the physicochemical properties and in vivo behavior of the radioligands, though not dramatically so. To wit, pretargeted PET and biodistribution experiments with huA33-TCO in a murine model of colorectal cancer revealed that [^64^Cu]­Cu-SarAr-PEG_10_-Tz yielded comparable tumoral uptake but higher tumor-to-background activity concentration ratios for most tissues compared to [^64^Cu]­Cu-SarAr-Tz. These superior tumor-to-healthy organ indices led us to interrogate the dosimetry, safety, and efficacy of pretargeted radioimmunotherapy using a ^67^Cu-labeled variant of the PEG_10_-bearing radioligand. Ultimately, we found that PRIT with [^67^Cu]­Cu-SarAr-PEG_10_-Tz exhibited a favorable safety profile and produced a dose-dependent therapeutic effect.

## Supplementary Material



## Notes

Reagents and general procedures; detailed experimental methods for chemical syntheses and bioconjugation reactions; NMR, mass spectrometry, radiolabeling, radio-iTLC, and stability data; blood half-life analysis; and tables of biodistribution data (PDF)

## Abbreviations

%ID/g: percent injected dose per gram of tissue
iTLC: instant thin layer chromatography
mAb: monoclonal antibody
MSKCC: Memorial Sloan Kettering Cancer Center
PET: positron emission tomography
PBS: phosphate buffered saline
DMSO: dimethyl sulfoxide
SE-HPLC: size exclusion high-performance liquid chromatography
TCO: trans-cyclooctene
Tz: tetrazine
RIT: radioimmunotherapy
PRIT: pretargeted radioimmunotherapy
RT: room temperature
PEG: polyethylene glycol
