# Supplementary material for: Pharmacokinetic Optimization of Radiocopper-Based Theranostic Pretargeting
Source: Mol Pharm. 2026 Jan 28;23(3):1749–57. doi: 10.1021/acs.molpharmaceut.5c01512 (PMC12958289; doi:10.1021/acs.molpharmaceut.5c01512)
Supplement: Supplementary file 1 [file mp5c01512_si_001.pdf]

Supporting Information

Pharmacokinetic Optimization of Radiocopper-Based Theranostic Pretargeting

Mike A. Cornejo[1,2,3], Zachary V. Samuels[1,2,3], Gina Dehlavi[1,2,4], Lukas Carter[5], Wei-Siang Mark Kao[1,3]
Emilia Strugala[1], Brian M. Zeglis[1,2,4,6,*]

[1]Department of Chemistry, Hunter College, The City University of New York, New York, NY, USA
[2]Ph.D. Program in Chemistry, Graduate Center of the City University of New York, New York, NY, USA
[3]Department of Radiology, Memorial Sloan Kettering Cancer Center, New York, NY, USA
[4]Ph.D. Program in Biochemistry, Graduate Center of the City University of New York, New York, NY, USA
[5]Department of Medical Physics, Memorial Sloan Kettering Cancer Center, New York, NY, USA
[6]Department of Radiology, Weill Cornell Medical College, New York, NY, USA

SUPPLEMENTAL METHODS

Longitudinal Therapy Study

Athymic nude mice bearing SW1222 xenografts were randomly sorted into 7 cohorts, which included three control cohorts and four experimental cohorts of 10 mice each.

Control cohorts

Mice were warmed gently using a heat lamp before intravenous tail vein injection. The first control cohort received only saline (50 μL), the second received only huA33-TCO (100 μg, 0.7 nmol, in 100 μL sterile saline), and the third received only [^{67}Cu]Cu-Sar-PEG$_{10}$-Tz (55.5 MBq, 0.7 nmol, in 100 μL sterile saline).

Radioimmunotherapy (RIT) cohort

For the preparation of the radioimmunoconjugate, huA33-TCO was mixed with a 10-fold excess of [^{67}Cu]Cu-SarAr-PEG$_{10}$-Tz and allowed to react for 10 min at room temperature. The mixture was purified using a PD-10 column, and radio-iTLC was used to check the purity of the radioimmunoconjugate with a mobile phase of 50 mM EDTA (pH 5.5). Mice were warmed gently using a heat lamp before the intravenous tail vein injection of [^{67}Cu]Cu-SarAr-PEG$_{10}$-huA33 (100 μg, 0.7 nmol, 18.5 MBq, in 100 μL sterile saline).

Pretargeted radioimmunotherapy (PRIT) cohort

Mice were warmed gently using a heat lamp before the intravenous tail vein injection of huA33-TCO (100 μg, 0.7 nmol, in 100 μL sterile saline). After 96 h, the same mice received a second injection of either 18.5 MBq, 37 MBq, or 55.5 MBq of [^{67}Cu]Cu-Sar-PEG$_{10}$-Tz (0.7 nmol in 100 μL sterile saline).

Tumor Measurements

Mice were monitored throughout the therapy study, and tumors were measured twice a week using calipers.

Blood analysis

Mice were anaesthetized with 2% isoflurane/oxygen gas mixture before collecting 30 μL of blood from the retro-orbital sinus using a micro-hematocrit capillary tube. The blood was quickly collected in a Minivette POCT collection tube coated with K3 EDTA (Braintree Scientific, Inc., Braintree, MA, USA). The samples were analyzed with HemaVet 950 (Drew Scientific, Inc., Miami Lakes, FL, USA).

FIGURES AND FIGURE CAPTIONS

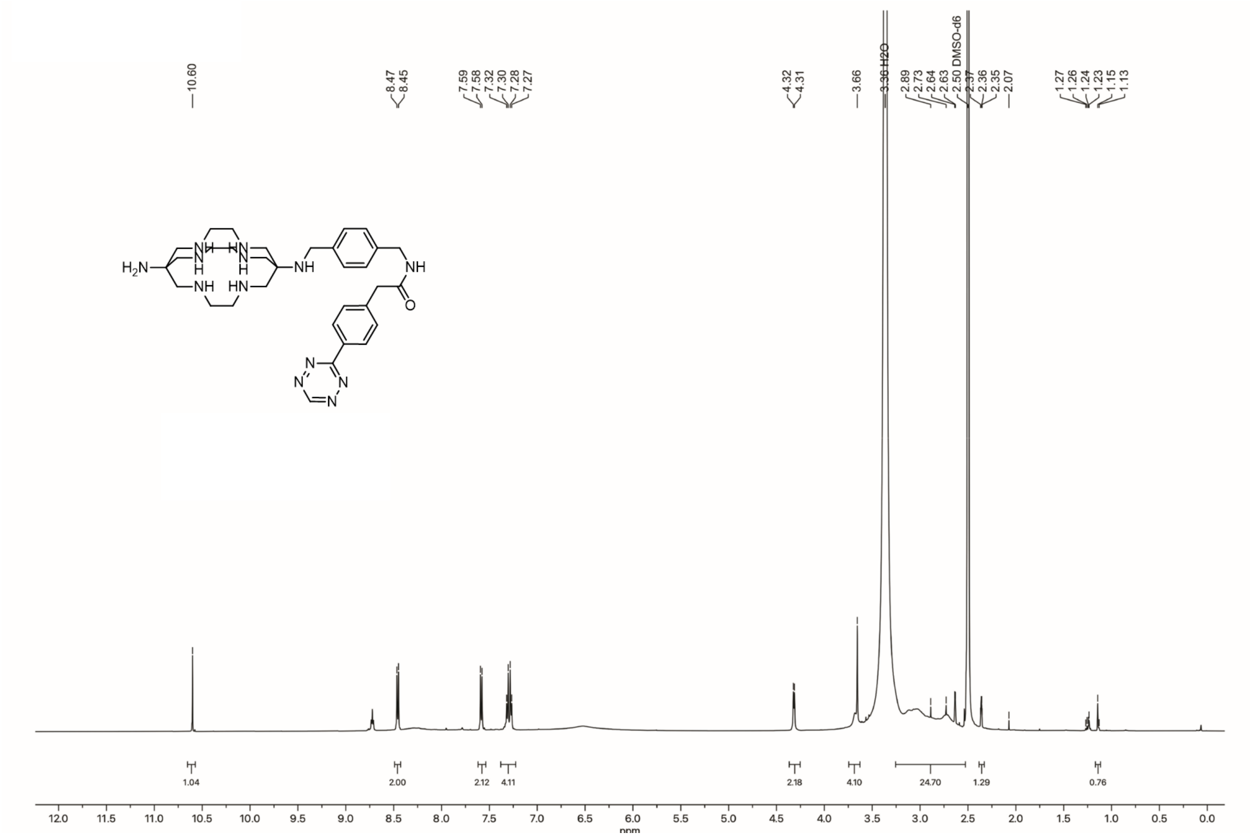

Figure S1. ^{1}H-NMR of SarAr-Tz (500 MHz, DMSO-d_6): δ = 10.60 (s, 1H), 8.47 (d, J = 5.0 Hz, 2H), 7.59 (d, J = 5.0 Hz, 2H), 7.32 (dd, J = 10.0, 20.0 Hz, 4H), 4.32 (d, J = 5.0 Hz, 2H), 3.66 (s, 4H), 3.2–2.6 (m, 24H).

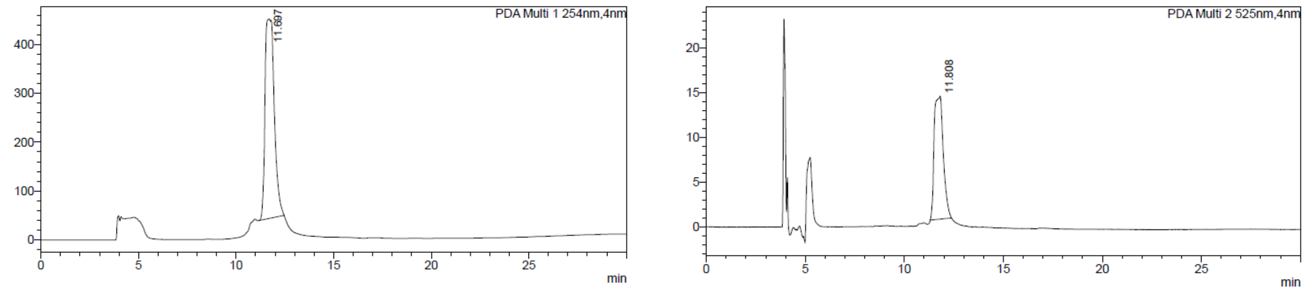

Figure S2. HPLC chromatograms of SarAr-Tz at 254 and 525 nm.

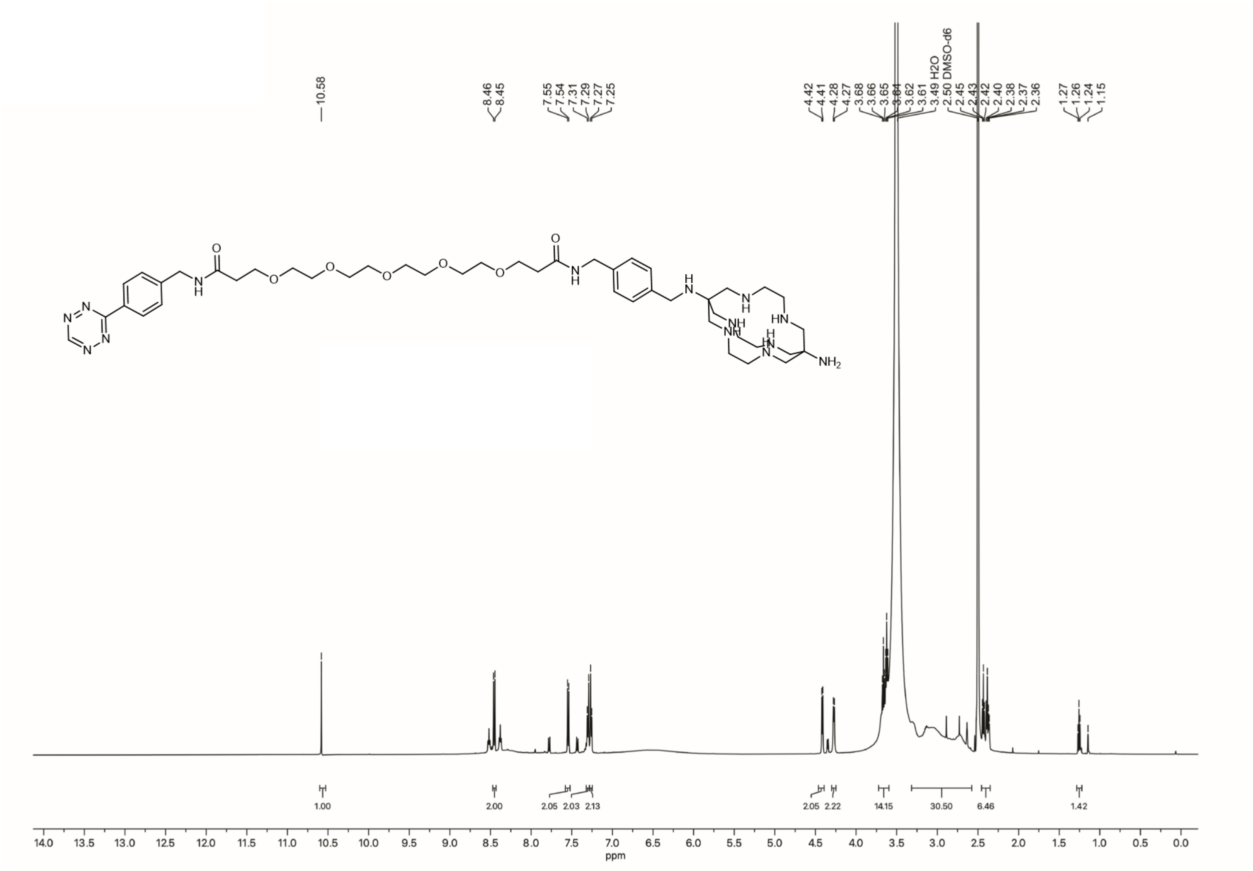

Figure S3. ^{1}H-NMR of SarAr-PEG$_5$-Tz (500 MHz, DMSO-d$_6$): δ = 10.50 (s, 1H), 8.46 (d, J = 5.0 Hz, 2H), 7.55 (d, J = 5.0 Hz, 2H), 7.31 (d, J = 10.0 Hz, 2H), 7.27 (d, J = 10.0 Hz, 2H), 4.42 (d, J = 5.0 Hz, 2H), 4.28 (d, J = 5.0 Hz, 2H), 3.68–3.60 (m, 14H), 3.35–2.55 (m, 30H), 2.45–2.35 (m, 6H), 1.26 (t, J = 5.0 Hz, 2H).

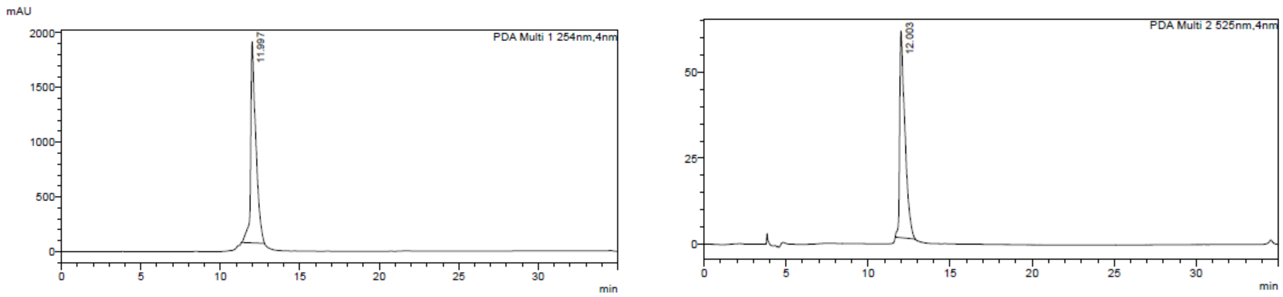

Figure S4. HPLC chromatograms of SarAr-PEG$_5$-Tz at 254 and 525 nm.

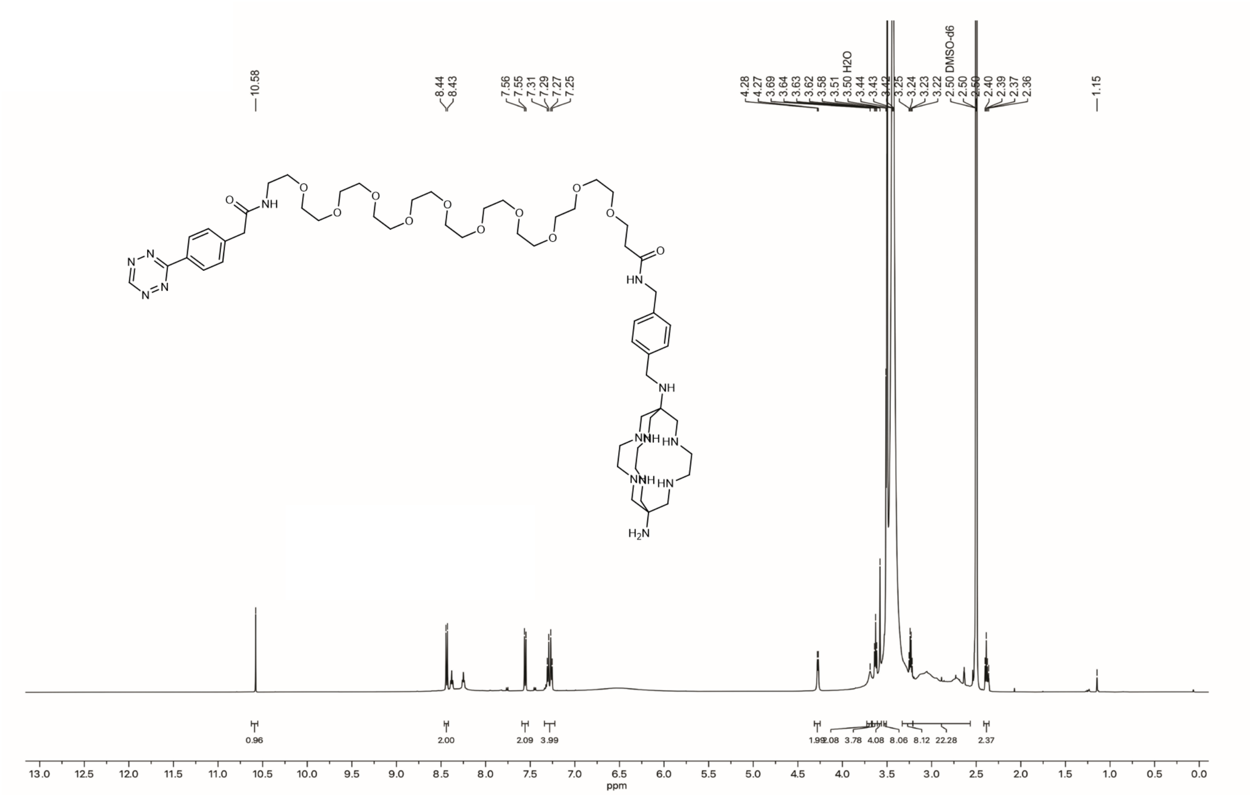

Figure S5. ^{1}H-NMR of SarAr-PEG$_{10}$-Tz (500 MHz, DMSO-d$_6$): δ = 10.58 (s, 1H), 8.44 (d, J = 10.0 Hz, 2H), 7.56 (d, J = 5.0 Hz, 2H), 7.27 (dd, J = 10.0, 20.0 Hz, 4H), 4.28 (d, J = 5.0 Hz, 2H), 3.69 (bs, 2H), 3.64 (d, J = 5.0 Hz, 4H), 3.58 (s, 4H), 3.51 (s, 8H), 3.25 (m, 8H), 3.20–2.55 (m, 22H), 2.40 (m, 2H).

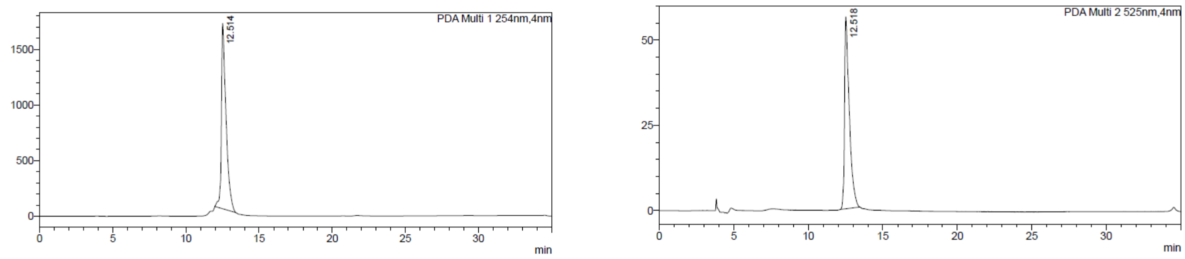

Figure S6. HPLC chromatograms of SarAr-PEG$_{10}$-Tz at 254 nm and 525 nm.

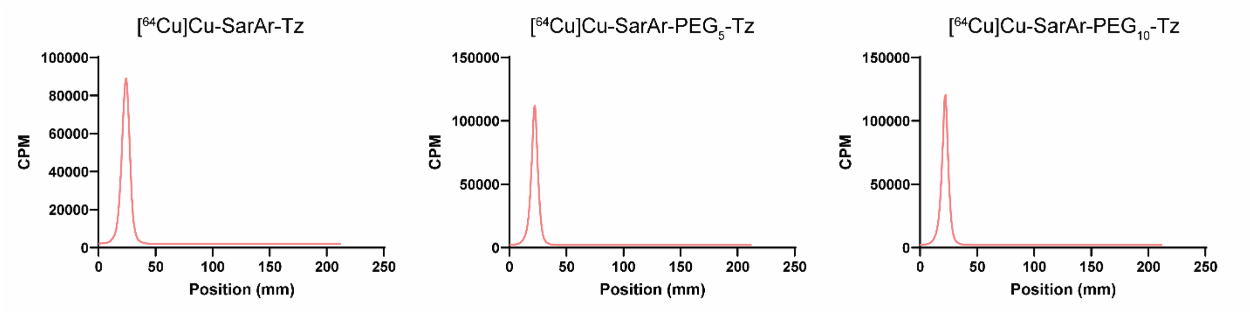

Figure S7. Radiolabeled iTLCs for the trio sarcophagine radioligands. Following the successful radiolabeling of the trio, radio-iTLCs were performed using EDTA (50 mM, pH 5.0) as the mobile phase. The trio exhibited >99% radiochemical purity.

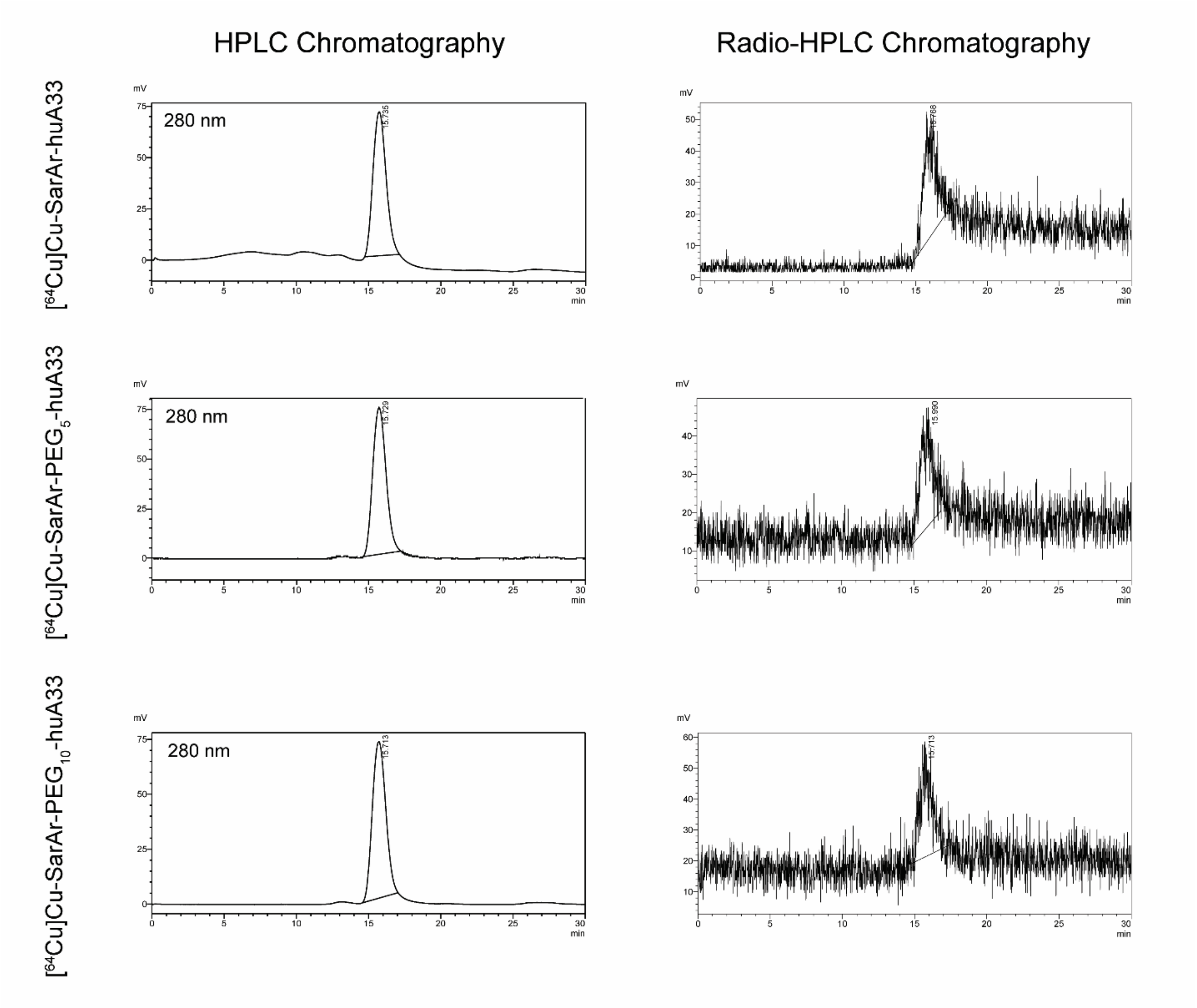

Figure S8. Size-exclusion HPLC for the click reaction. Following the radiosynthesis of the three Tz ligands, a test click reaction was performed by combining huA33-TCO with [^{64}Cu]Cu-SarAr-Tz, [^{64}Cu]Cu-SarAr-PEG$_5$-Tz, or [^{64}Cu]Cu-SarAr-PEG$_{10}$-Tz. After 10 min, the mixture was purified using a PD-10 column, and a small sample of the purified radioimmunoconjugate was injected into radio-SE HPLC, which was monitored using the 280 nm and radioactivity channels.

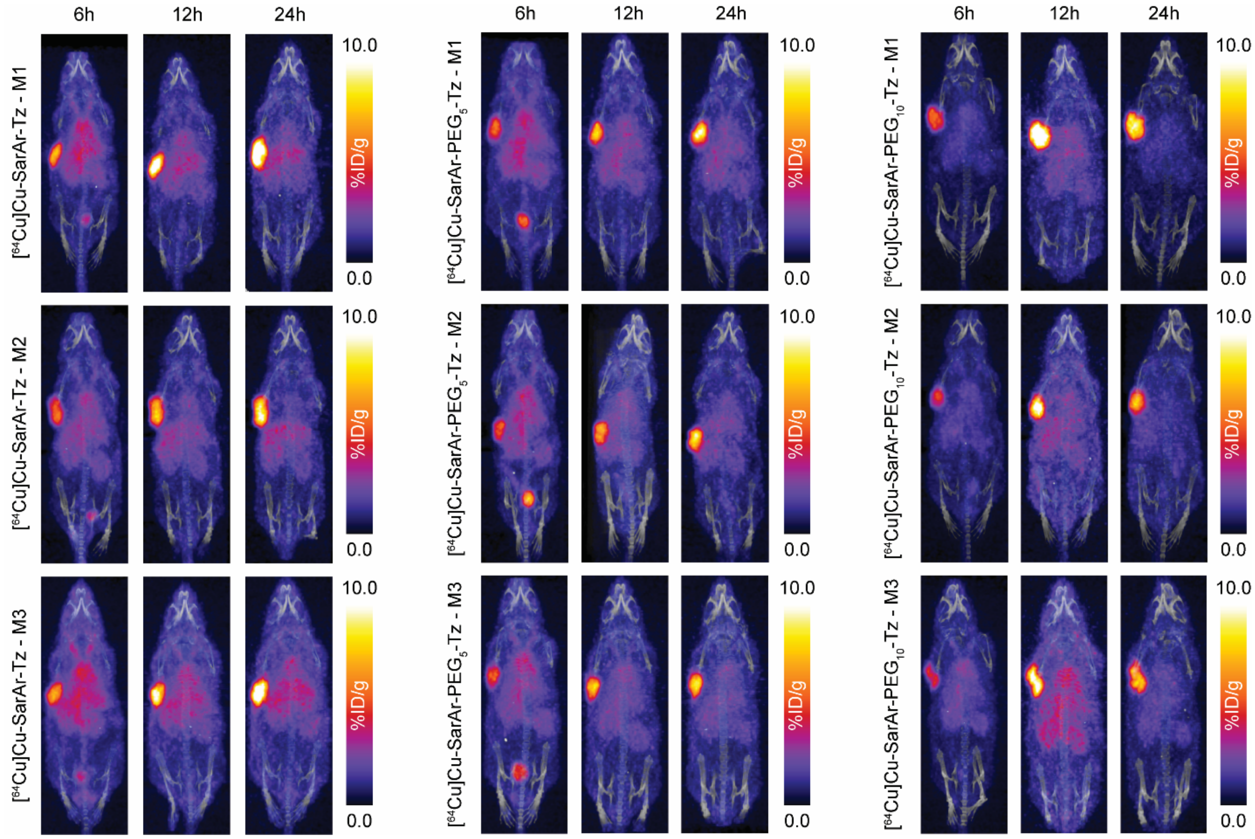

Figure S9. Pretargeted PET Images. Athymic nude mice received a first injection of huA33-TCO (100 µg, 0.7 nmol). After a period of 96 h, the same mice received a second injection of [^{64}Cu]Cu-SarAr-Tz, [^{64}Cu]Cu-SarAr-PEG$_5$-Tz, or [^{64}Cu]Cu-SarAr-PEG$_{10}$-Tz (11.1 – 12.9 MBq, 0.7 nmol). Images were acquired 6, 12, and 24 h after the administration of the radioligands.

TABLES AND TABLE LEGENDS

	**Cy3-Tz/huA33-TCO**
**DOL (TCO/mAb)**	5.4 ± 0.8

Table S1. Degree of labeling results for the modification of huA33 with TCO.

Organs	[^{64}Cu]Cu-SarAr-Tz	[^{64}Cu]Cu-SarAr-PEG$_5$-Tz	[^{64}Cu]Cu-SarAr-PEG$_{10}$-Tz
Blood	3.0 ± 0.5	2.8 ± 0.2	1.9 ± 0.3
Tumor	12.9 ± 2.5	10.2 ± 0.7	8.7 ± 1.4
Heart	1.3 ± 0.2	1.2 ± 0.1	0.8 ± 0.0^{2}
Lungs	2.4 ± 0.2	2.3 ± 0.2	1.6 ± 0.1
Liver	3.0 ± 0.3	2.1 ± 0.2	1.3 ± 0.1
Spleen	1.9 ± 0.1	1.7 ± 0.2	1.0 ± 0.1
Pancreas	0.7 ± 0.0^{0}	0.6 ± 0.1	0.4 ± 0.1
Stomach	0.4 ± 0.0^{0}	0.4 ± 0.1	0.2 ± 0.0^{2}
Sm Int.	0.6 ± 0.1	0.5 ± 0.1	0.3 ± 0.0^{2}
Lg Int.	0.4 ± 0.1	0.4 ± 0.1	0.3 ± 0.1
Kidney	3.4 ± 0.1	3.0 ± 0.4	2.1 ± 0.2
Muscle	0.5 ± 0.1	0.4 ± 0.1	0.3 ± 0.0^{3}
Bone	0.7 ± 0.1	0.7 ± 0.0^{1}	0.5 ± 0.1
Skin	2.1 ± 0.5	1.4 ± 0.1	1.1 ± 0.2
Tail	1.3 ± 0.6	0.7 ± 0.1	0.5 ± 0.1

Table S2. Activity concentration data from *in vivo* pretargeting in mice bearing SW1222 xenografts. Athymic nude mice received a first injection of huA33-TCO (100 µg, 0.7 nmol). After a period of 96 h, the same mice received a second injection of [^{64}Cu]Cu-SarAr-Tz, [^{64}Cu]Cu-SarAr-PEG$_5$-Tz, or [^{64}Cu]Cu-SarAr-PEG$_{10}$-Tz (11.1 – 12.95 MBq, 0.7 nmol). After 24 h, the mice were euthanized; relevant tissues were collected, washed, and weighed; and the amount of radioactivity in each tissue was measured using a gamma counter. Values are in units of %ID/g ± standard deviations.

Organs	[^{64}Cu]Cu-SarAr-Tz	[^{64}Cu]Cu-SarAr-PEG$_5$-Tz	[^{64}Cu]Cu-SarAr-PEG$_{10}$-Tz
Blood	4.3 ± 1.1	3.6 ± 0.4	4.5 ± 1.0
Tumor	1	1	1
Heart	9.8 ± 2.6	8.7 ± 1.3	10.4 ± 1.7
Lungs	5.3 ± 1.8	4.5 ± 0.5	5.5 ± 0.9
Liver	4.2 ± 0.9	4.9 ± 0.6	6.8 ± 1.3
Spleen	6.6 ± 1.4	6.0 ± 0.8	8.6 ± 1.7
Pancreas	17.2 ± 3.6	15.8 ± 2.1	19.7 ± 6.3
Stomach	29.7 ± 6.6	27.0 ± 9.0	50.0 ± 9.8
Sm Int	21.1 ± 5.1	22.0 ± 3.5	27.0 ± 4.8
Lg Int	30.6 ± 7.2	23.6 ± 3.1	30.8 ± 8.0
Kidney	3.8 ± 0.8	3.4 ± 0.5	4.0 ± 0.8
Muscle	27.8 ± 6.6	23.9 ± 3.1	32.6 ± 6.2
Bone	17.2 ± 4.1	14.4 ± 1.0	20.4 ± 5.4
Skin	6.0 ± 1.7	7.4 ± 0.7	8.2 ± 2.0
Tail	9.6 ± 4.8	13.5 ± 2.5	18.2 ± 4.2

Table S3. Tumor-to-healthy organ activity concentration ratios from *in vivo* pretargeting in mice bearing SW1222 xenografts. Athymic nude mice received a first injection of huA33-TCO (100 µg, 0.7 nmol). After a period of 96 h, the same mice received a second injection of [^{64}Cu]Cu-SarAr-Tz, [^{64}Cu]Cu-SarAr-PEG$_5$-Tz, or [^{64}Cu]Cu-SarAr-PEG$_{10}$-Tz (11.1 – 12.95 MBq, 0.7 nmol). After 24 h, the mice were euthanized; relevant tissues were collected, washed, and weighed; and the amount of radioactivity in each tissue was measured using a gamma counter. Values are unitless ± standard deviations.

**Organs**	**%ID/g** *$[^{64}Cu]$Cu-SarAr-Tz*	**%ID/g** *$[^{64}Cu]$Cu-SarAr-PEG_5-Tz*	**p Value**	**Significance**
Blood	3.0 ± 0.5	2.8 ± 0.2	0.6	No
Tumor	12.9 ± 2.5	10.2 ± 0.7	0.2	No
Heart	1.3 ± 0.2	1.2 ± 0.1	0.5	No
Lungs	2.4 ± 0.2	2.3 ± 0.2	0.4	No
Liver	3.0 ± 0.3	2.1 ± 0.2	0.01	Yes
Spleen	1.9 ± 0.1	1.7 ± 0.2	0.1	No
Pancreas	0.7 ± 0.0^{4}	0.6 ± 0.1	0.1	No
Stomach	0.4 ± 0.0^{4}	0.4 ± 0.1	0.6	No
Sm Int	0.6 ± 0.1	0.5 ± 0.1	0.06	No
Lg Int	0.4 ± 0.1	0.4 ± 0.1	0.8	No
Kidneys	3.4 ± 0.1	3.0 ± 0.4	0.2	No
Muscle	0.5 ± 0.1	0.4 ± 0.1	0.5	No
Bone	0.7 ± 0.1	0.7 ± 0.0^{1}	0.5	No
Skin	2.1 ± 0.5	1.4 ± 0.1	0.1	No
Tail	1.3 ± 0.6	0.7 ± 0.1	0.2	No

Table S4. Statistical comparison of the uptake values produced by pretargeting with $[^{64}Cu]$Cu-SarAr-Tz and $[^{64}Cu]$Cu-SarAr-PEG_5-Tz. The p value between each set of %ID/g values was obtained using an unpaired t-test with Welch's correction in GraphPad software.

Organs	**%ID/g**<br>*[^{64}Cu]Cu-SarAr-Tz*	**%ID/g**<br>*[^{64}Cu]Cu-SarAr-PEG$_{10}$-Tz*	**p Value**	**Significance**
Blood	3.0 ± 0.5	1.9 ± 0.3	0.03	Yes
Tumor	12.9 ± 2.5	8.7 ± 1.4	0.08	No
Heart	1.3 ± 0.2	0.8 ± 0.0^{2}	0.07	No
Lungs	2.4 ± 0.2	1.6 ± 0.1	0.01	Yes
Liver	3.0 ± 0.3	1.3 ± 0.1	0.003	Yes
Spleen	1.9 ± 0.1	1.0 ± 0.1	0.0005	Yes
Pancreas	0.7 ± 0.0^{4}	0.4 ± 0.1	0.04	Yes
Stomach	0.4 ± 0.0^{4}	0.2 ± 0.0^{2}	0.002	Yes
Sm Int	0.6 ± 0.1	0.3 ± 0.0^{2}	0.02	Yes
Lg Int	0.4 ± 0.1	0.3 ± 0.1	0.04	Yes
Kidneys	3.4 ± 0.1	2.1 ± 0.2	0.003	Yes
Muscle	0.5 ± 0.1	0.3 ± 0.0^{3}	0.01	Yes
Bone	0.7 ± 0.1	0.5 ± 0.1	0.02	Yes
Skin	2.1 ± 0.5	1.1 ± 0.2	0.04	Yes
Tail	1.3 ± 0.6	0.5 ± 0.1	0.1	No

Table S5. Statistical comparison of the uptake values produced by pretargeting with [^{64}Cu]Cu-SarAr-Tz and [^{64}Cu]Cu-SarAr-PEG$_{10}$-Tz. The p value between each set of %ID/g values was obtained using an unpaired t-test with Welch's correction in GraphPad software.

Organs	**%ID/g** *$[^{64}$Cu]Cu-SarAr-PEG$_5$-Tz*	**%ID/g** *$[^{64}$Cu]Cu-SarAr-PEG$_{10}$-Tz*	p Value	Significance
Blood	2.8 ± 0.2	1.9 ± 0.3	0.02	Yes
Tumor	10.2 ± 0.7	8.7 ± 1.4	0.2	No
Heart	1.2 ± 0.1	0.8 ± 0.0[2]	0.06	No
Lungs	2.3 ± 0.2	1.6 ± 0.1	0.008	Yes
Liver	2.1 ± 0.2	1.3 ± 0.1	0.01	Yes
Spleen	1.7 ± 0.2	1.0 ± 0.1	0.008	Yes
Pancreas	0.6 ± 0.1	0.4 ± 0.1	0.08	No
Stomach	0.4 ± 0.1	0.2 ± 0.0[2]	0.09	No
Sm Int	0.5 ± 0.1	0.3 ± 0.0[2]	0.04	Yes
Lg Int	0.4 ± 0.1	0.3 ± 0.1	0.03	Yes
Kidneys	3.0 ± 0.4	2.1 ± 0.2	0.03	Yes
Muscle	0.4 ± 0.1	0.3 ± 0.0[3]	0.01	Yes
Bone	0.7 ± 0.0[1]	0.5 ± 0.1	0.04	Yes
Skin	1.4 ± 0.1	1.1 ± 0.2	0.07	No
Tail	0.7 ± 0.1	0.5 ± 0.1	0.05	Yes

Table S6. Statistical comparison of the uptake values produced by pretargeting with $[^{64}$Cu]Cu-SarAr-PEG$_5$-Tz and $[^{64}$Cu]Cu-SarAr-PEG$_{10}$-Tz. The p value between each set of %ID/g values was obtained using an unpaired t-test with Welch’s correction in GraphPad software.

Organs	Tumor-to-Organ Ratio *[^{64}Cu]Cu-SarAr-Tz*	Tumor-to-Organ Ratio *[^{64}Cu]Cu-SarAr-PEG$_5$-Tz*	p Value	Significance
Blood	4.3 ± 1.1	3.6 ± 0.4	0.4	No
Tumor	1	1	-	-
Heart	9.8 ± 2.6	8.7 ± 1.3	0.5	No
Lungs	5.3 ± 1.8	4.5 ± 0.5	0.3	No
Liver	4.2 ± 0.9	4.9 ± 0.6	0.4	No
Spleen	6.6 ± 1.4	6.0 ± 0.8	0.5	No
Pancreas	17.2 ± 3.6	15.8 ± 2.1	0.6	No
Stomach	29.7 ± 6.6	27.0 ± 9.0	0.7	No
Sm Int	21.1 ± 5.1	22.0 ± 3.5	0.8	No
Lg Int	30.6 ± 7.2	23.6 ± 3.1	0.2	No
Kidneys	3.8 ± 0.8	3.4 ± 0.5	0.5	No
Muscle	27.8 ± 6.6	23.9 ± 3.1	0.4	No
Bone	17.2 ± 4.1	14.4 ± 1.0	0.4	No
Skin	6.0 ± 1.7	7.4 ± 0.7	0.3	No
Tail	9.6 ± 4.8	13.5 ± 2.5	0.3	No

Table S7. Statistical comparison of the tumor-to-healthy tissue activity concentration ratios produced by pretargeting with [^{64}Cu]Cu-SarAr-Tz and [^{64}Cu]Cu-SarAr-PEG$_5$-Tz. The p value between each set of ratios was obtained using a multiple unpaired t-test via GraphPad software.

Organs	Tumor-to-Organ Ratio *[^{64}Cu]Cu-SarAr-Tz*	Tumor-to-Organ Ratio *[^{64}Cu]Cu-SarAr-PEG$_{10}$-Tz*	p Value	Significance
Blood	4.3 ± 1.1	4.5 ± 1.0	0.8	No
Tumor	1	1	-	-
Heart	9.8 ± 2.6	10.4 ± 1.7	0.8	No
Lungs	5.3 ± 1.8	5.5 ± 0.9	0.9	No
Liver	4.2 ± 0.9	6.8 ± 1.3	0.06	No
Spleen	6.6 ± 1.4	8.6 ± 1.7	0.2	No
Pancreas	17.2 ± 3.6	19.7 ± 6.3	0.6	No
Stomach	29.7 ± 6.6	50.0 ± 9.8	0.05	Yes
Sm Int	21.1 ± 5.1	27.0 ± 4.8	0.2	No
Lg Int	30.6 ± 7.2	30.8 ± 8.0	1.0	No
Kidneys	3.8 ± 0.8	4.0 ± 0.8	0.7	No
Muscle	27.8 ± 6.6	32.6 ± 6.2	0.4	No
Bone	17.2 ± 4.1	20.4 ± 5.4	0.5	No
Skin	6.0 ± 1.7	8.2 ± 2.0	0.2	No
Tail	9.6 ± 4.8	18.2 ± 4.2	0.08	No

Table S8. Statistical comparison of the tumor-to-healthy tissue activity concentration ratios produced by pretargeting with [^{64}Cu]Cu-SarAr-Tz and [^{64}Cu]Cu-SarAr-PEG$_{10}$-Tz. The p value between each set of ratios was obtained using a multiple unpaired t-test via GraphPad software.

Organs	**Tumor-to-Organ Ratio** *[^{64}Cu]Cu-SarAr-PEG$_5$-Tz*	**Tumor-to-Organ Ratio** *[^{64}Cu]Cu-SarAr-PEG$_{10}$-Tz*	**P Values**	**Significance**
Blood	3.6 ± 0.4	4.5 ± 1.0	0.2	No
Tumor	1	1	-	-
Heart	8.7 ± 1.3	10.4 ± 1.7	0.2	No
Lungs	4.5 ± 0.5	5.5 ± 0.9	0.2	No
Liver	4.9 ± 0.6	6.8 ± 1.3	0.1	No
Spleen	6.0 ± 0.8	8.6 ± 1.7	0.1	No
Pancreas	15.8 ± 2.1	19.7 ± 6.3	0.4	No
Stomach	27.0 ± 9.0	50.0 ± 9.8	0.04	Yes
Sm Int	22.0 ± 3.5	27.0 ± 4.8	0.2	No
Lg Int	23.6 ± 3.1	30.8 ± 8.0	0.2	No
Kidneys	3.4 ± 0.5	4.0 ± 0.8	0.3	No
Muscle	23.9 ± 3.1	32.6 ± 6.2	0.1	No
Bone	14.4 ± 1.0	20.4 ± 5.4	0.2	No
Skin	7.4 ± 0.7	8.2 ± 2.0	0.6	No
Tail	13.5 ± 2.5	18.2 ± 4.2	0.2	No

Table S9. Statistical comparison of the tumor-to-healthy tissue activity concentration ratios produced by pretargeting with [^{64}Cu]Cu-SarAr- PEG$_5$-Tz and [^{64}Cu]Cu-SarAr-PEG$_{10}$-Tz. The p value between each set of ratios was obtained using a multiple unpaired t-test via GraphPad software.
